# Salivary Redox Biomarkers in Selected Neurodegenerative Diseases

**DOI:** 10.3390/jcm9020497

**Published:** 2020-02-12

**Authors:** Mateusz Maciejczyk, Anna Zalewska, Karolina Gerreth

**Affiliations:** 1 Department of Hygiene, Epidemiology and Ergonomics, Medical University of Bialystok, 2C Adama Mickiewicza Street, 15-022 Bialystok, Poland; mat.maciejczyk@gmail.com; 2 Experimental Dentistry Laboratory, Medical University of Bialystok, 24A Marii Sklodowskiej-Curie Street, 15-276 Bialystok, Poland; azalewska426@gmail.com; 3 Department of Risk Group Dentistry, Chair of Pediatric Dentistry, Poznan University of Medical Sciences, 70 Bukowska Street, 60-812 Poznan, Poland

**Keywords:** neurodegenerative diseases (NDDs), saliva, oxidative stress, salivary biomarkers

## Abstract

Neurodegenerative diseases (NDDs), such as Alzheimer’s disease, Parkinson’s disease, and Huntington’s disease, are disorders, which cause irreversible and progressive deterioration of the central nervous system. The pathophysiology of NDDs is still not fully explained; nevertheless, oxidative stress is considered as a critical mediator of cerebral degeneration, brain inflammation, as well as neuronal apoptosis. Therefore, it is not surprising that redox biomarkers are increasingly used in the diagnosis of neurodegenerative diseases. As saliva is a very easy to obtain bioliquid, it seems promising to use this biomaterial in the diagnosis of NDDs. Saliva collection is easy, cheap, stress-free, and non-infectious, and it does not require the help of a specialised medical personnel. Additionally, the concentrations of many salivary redox biomarkers correlate with their content in blood serum as well as the degree of disease progression, which makes them non-invasive indicators of NDDs. This paper reviews the latest knowledge concerning the use of salivary redox biomarkers in the diagnosis and prognosis of selected neurodegenerative diseases.

## 1. Introduction

Currently, salivary biomarkers are used for the diagnosis, prognosis, and monitoring of numerous disorders in different fields of medicine. This is possible due to the content of organic and inorganic substances that are found in the saliva [1]. 

The most researched salivary biomarkers include immunoglobulins (e.g., IgA), matrix metalloproteinases [2], mucins [3], cytokines [4], human α- and β-defensins [5], as well as oxidative stress products [6]. Literature data showed the use of salivary biomarkers in the diagnosis of periodontitis, oral cancers [7,8,9,10], and Sjögren’s syndrome [11,12]. Interestingly, microorganisms, electrolytes, proteins, and peptides that are derived from saliva are utilized for dental caries diagnosis [13,14]. 

Furthermore, salivary biomarkers have been recognized as those that could be used in early diagnosis of some systemic disorders, such as diabetes mellitus [15], cardiovascular diseases [16], as well as breast cancer [17,18]. However, among all of the biomarkers measured in saliva, it is the indicators of oxidative stress that are becoming increasingly popular. Interestingly, the results of recent studies indicate the high diagnostic value of salivary redox parameters in the diagnosis of obesity [19,20], insulin resistance [21,22], chronic kidney disease [23,24], but also neurodegenerative diseases (NDDs) [1,25,26]. Indeed, salivary antioxidants, as well as products of protein, lipid, and DNA oxidation, are proposed as potential diagnostic biomarkers. 

There are no studies reviewing the latest reports in the field of salivary redox diagnostics of NDDs. Therefore, this paper aims to provide a state-of-the-art summary of the use of salivary biomarkers of oxidative stress for the diagnosis and monitoring of selected neurodegenerative diseases. 

Studies that were included in the present paper were identified from searches of PubMed, Web of Science, and Google Scholar. We included the case-control studies with human subjects, as well as major relevant review papers. The articles selected for this paper were published in English between 1988 and 2020. To find all of the relevant papers, the databases were searched while using the keywords: “oxidative stress”, “OS”, “neurodegenerative disease”, “NDD”, “antioxidant”, “biomarker”, ”saliva”, and “Alzheimer’s disease”, “dementia”, “Parkinson’s disease”, “Huntington’s disease”, as well as “amyotrophic lateral sclerosis” in various combinations. However, only seven original papers were found that took into consideration salivary redox biomarkers of NDDs diagnostics, including Alzheimer’s disease, dementia, and Parkinson’s disease. 

## 2. Saliva: Composition and Diagnostic Importance

Saliva is an essential factor that affects the homeostasis of the oral cavity [27,28]. It is produced and secreted by the small as well as large salivary glands into the oral cavity [27]. The saliva has various physiological functions, such as providing the proper environment for teeth and mucosa, protecting against mechanical damage of the tissues, as well as numerous chemical and biological substances [1,29,30]. Because of the presence of, e.g., lysozyme, lactoferrin, and immunoglobulins, it has defensive properties against pathogens, like some viruses, bacteria, or fungi [1,31]. Moreover, this biofluid plays multiple roles concerning the taste, mastication, bolus formation, swallowing, and initial digestive processes being in progress in the upper parts of the gastrointestinal tract [29]. 

In saliva, numerous inorganic components could be found, such as HCO_3_^-^, F^-^, I^-^, Mg^2+^, Na^+^, Cl^-^, Ca^2+^, and K^+^. Besides, it also contains many organic biomolecules: urea, uric acid, ammonia, glucose, glycolipids, triglycerides, fatty acids, steroid hormones, mucins, amylase, lectins, glycoproteins, lysozyme, salivary peroxidase, as well as lactoferrin [32]. Moreover, this fluid contains more than 700 microorganisms that are related to systemic as well as oral diseases.

The antioxidants are one of the most important components of saliva and, therefore, the oral cavity is the first protective barrier against systemic oxidative stress [26,33,34]. The salivary antioxidant systems are responsible for limiting the over-accumulation of free radicals. They are both enzymatic, which include superoxide dismutase (SOD), catalase (CAT), thioredoxin reductase (TR), and salivary peroxidase (Px), as well as non-enzymatic (e.g., uric acid, α-tocopherol (vitamin E), reduced glutathione (GSH), ascorbic acid (vitamin C), coenzyme Q, melatonin, flavonoids, selenium, carotenoids, or lipoic acid) [35]. However, the most important oral antioxidant is uric acid, which accounts for over 70–80% of the antioxidant capacity of saliva [22,33,34].

Currently, saliva is considered as a non-invasive and ideal diagnostic material in comparison to the blood, where there might be some risk of patients/medical professionals’ infection with pathogens, like HCV, HBV, or HIV [27,30]. Moreover, it could be acquired without any discomfort in comparison to blood or cerebrospinal fluid (CSF) collection [27]. Therefore, biomarkers from saliva might be easily gained, especially in small children and even newborn [36]. The samples can be even self-collected by the patients at home or with the help of a caregiver, and easily store in the refrigerator until diagnostics [37]. The fluid is also accessible due to its close relationship with plasma [38]. Indeed, many organic/inorganic substances, as well as some drugs, are excreted into the saliva, and their salivary level correlates with the blood content [1,24,25,26,28,30]. The passage of the substance into the saliva depends on the type of mechanism and occurs *via* the intracellular or extracellular route. The intracellular pathway includes passive transport (diffusion or filtration), facilitated diffusion, active transport, as well as pinocytosis. On the other hand, the extracellular route involves ultrafiltration or transport through damaged membranes [22,33,34]. Interestingly, saliva-penetrating compounds include hormones, electrolytes, and drugs, as well as antioxidants and oxidative damage products [1,22,24,25,26,27,28,32]. 

At present, saliva is commonly used as a diagnostic tool in drug or alcohol abuse [39,40,41,42]. Interestingly, the fluid derived from the lip prints or bite marks of a victim, at the crime scene, could be used for the identification of the accused due to cellular and serological analysis (e.g. salivary DNA) [38]. Moreover, in forensic medicine, saliva is also useful for screening samples containing species-specific DNA profiles for unknown animal identification [43]. The screening of heavy metal poisoning and other toxic substances in saliva samples is practicable, especially when the blood is not available to obtain due to different reasons, mostly in small children [44,45]. Furthermore, the salivary biomarkers provide vital information regarding the level of stress, even in critically ill pediatric patients [46]. 

Salivary diagnostics has also many limitations despite the undoubted advantages. The level of salivary biomarkers might vary depending on age, sex, salivary flow, systemic hydration status, as well as local changes in the oral environment (e.g. periodontal disease and oral mucosa disease). There are also no reference values for all parameters that were measured in saliva, including, especially, salivary redox biomarkers [24,27,32].

## 3. Neurodegenerative Diseases (NDDs)

NDDs, such as Alzheimer’s disease (AD), Parkinson’s disease (PD), Huntington’s disease (HD), and amyotrophic lateral sclerosis (ALS), are disorders that are characterized by a loss of selectively vulnerable neurons that are associated with distinct progressive involvement of functional systems [47,48,49,50]. Finally, NDDs affect memory, cognition, or motor skills. However, the pathophysiology of neurodegenerative diseases is still not thoroughly explained [51,52,53]. The typical feature includes the deposition of proteins that show altered physicochemical properties in the peripheral organs as well as in the brain, in both intracellular (neurons or glial cells) and extracellular locations [49,54]. The proteins that are involved in such neuropathologies are α-synuclein, amyloid-β (Aβ), the microtubule-associated protein tau, prion protein (PrP), transactive response (TAR) DNA-binding protein 43 (TDP-43), FET proteins (include the fused-in sarcoma (FUS), Ewing sarcoma RNA-binding protein 1 (EWSR1), and TATA-binding protein-associated factor15 (TAF15)), and proteins that are associated with hereditary disorders (proteins encoded by genes linked to neurologic trinucleotide repeat disorders, neuroserpin, ferritin-related neurodegenerative diseases, and familial cerebral amyloidoses) [47,49,50]. 

A group of NDDs causes dementia in patients. Alzheimer’s disease is considered to be the most common form of dementia and it constitutes up to 80% of all dementia cases globally [35,55]. According to the World Health Organization (WHO), in 2015, the condition affected 47 million people worldwide, which is approximately 5% of the elderly population [56]. Alzheimer’s disease-related dementias are classified as Alzheimer’s dementia (AD), frontotemporal dementia (FTD), dementia with Lewy bodies (DLB), vascular dementia (VaD), as well as mixed dementias (MxD) [57]. The main signs of AD pathogenesis are the existence of tau neurofibrillary tangles (NFTs) and amyloid-β (Aβ) plaques, which lead to synaptic loss [58]. With time, this pathology causes cognitive deterioration with impaired vision, speech, behavior, and, finally, leads to death [53,58,59]. 

Parkinson’s disease is considered to be the second most prevalent neurodegenerative disease [53]. The world’s population suffering from PD in 1990 was estimated at 2.5 million individuals in comparison to as much as 6.1 million in 2016 [60]. The characteristic feature of the disorder is dopaminergic neuron loss in the substantia nigra pars compacta of the brain, which consequently causes dopamine depletion in the striatum [53,61]. Unfortunately, approximately 70% of the dopaminergic neurons in the brain’s nigrostriatal pathway of the individuals are lost before the occurrence of the characteristic motor symptoms in the patient (such as postural instability, tremor, rigidity, and bradykinesia, i.e., slowness of movement) [62,63]. Additionally, abnormal aggregation of α-synuclein is also observed in surviving neurons [61,62]. 

Importantly, oxidative stress (OS) has been suggested as one of the critical elements in the pathogenesis of neurodegenerative disorders, which is caused by free radicals or other reactive oxygen (ROS)/nitrogen (RNS) species [51,53]. 

## 4. Oxidative Stress in Neurodegenerative Diseases

Living cells continually generate free radicals through respiratory reactions in mitochondria. At the low or moderate level, ROS and RNS have been indicated to mediate the induction of mitogenic response, the regulation of signal transduction, as well as the involvement in defense against infectious agents [35,64,65]. For example, long-term potentiation (LTP) through glutamate-dependent mechanisms is promoted by ROS-generated nitric oxide and carbon monoxide [64]. A gentle balance between harmful and beneficial effects of free radicals is a vital feature of living organisms that is maintained by mechanisms called “redox regulation” [66]. 

OS is caused by the imbalance between the formation of ROS/RNS and the antioxidant balance of the body [52,66,67]. ROS contain such species as hydrogen peroxide (H_2_O_2_), superoxide anion (O_2_^−^), and hydroxyl radical (OH), whereas RNS include peroxynitrite (ONOO^−^) and nitric oxide (NO) [68]. It was shown that OS might induce cellular damage, mitochondrial dysfunction, and the deterioration of the DNA repair system. Excessive free radicals can freely cross through the plasma membrane, cause destruction of the cell membrane via lipid peroxidation, modify structural and signal proteins leading to misfolding and aggregation, as well as oxidize DNA/RNA [35]. The damage of cell components through the oxidation process could be observed as an increase of oxidative-modified nucleic acids (8-OHdG—8-hydroxy-2-deoxyguanosine), lipids (8-isop—8-isoprostanes), and proteins (AOPP—advanced oxidation protein products as well as AGE—advanced glycation end products) [25]. The others markers might also be observed in the brains of individuals with AD and mild cognitive impairment, such as TBARS (thiobarbituric acid reactive substances), PUFA breakdown products, 4-HNE (4-hydroxy-2-nonenal), 3-NT (3-nitrotyrosine), as well as protein carbonyls [69].

Such actions play the main role in the acceleration of the aging process and the occurrence of NDDs. In the aging organism, a higher level of OS is observed due to of long-term exposure to ROS and inadequate defense mechanisms in the brain. Interestingly, ROS might modify different molecules within the cell, including proteins that were proven to be involved in neurodegenerative diseases [58]. Oxidative stress, as caused by ROS overproduction, creates an environment that is translationally, transcriptionally, and epigenetically favorable for Aβ (amyloid-β) production [58]. 

Cellular ROS are essentially generated by both sources, i.e., endogenous (nicotinamide adenine dinucleotide phosphate (NADPH) oxidase (NOX), cytochrome P450 from endoplasmic reticulum (ER), xanthine oxidase (XO), and flavin oxidases from peroxisomes), as well as exogenous (e.g., some drugs, ionizing radiation, ultraviolet, chemicals, and toxins play vital role in such process) [52]. Additionally, redox-active metal ions could catalyze the production of ROS when attached to the amyloid-β (Aβ) [70]. Oligomers, which are formed by amyloid β, could increase ROS production in mitochondria and regulate the action of alcohol dehydrogenase, which binds α-ketoglutarate dehydrogenase and amyloid β. They also increase the formation of hydrogen peroxide and activate NOX that is the primary source of free radicals in the cell [65,71]. 

It is needless to say that the brain is undoubtedly one of the most metabolically active parts of the body, and it is susceptible to redox imbalance and cellular oxidative damage [52,64,65]. On one hand, this organ has an elevated oxygen demand. On the other, the high levels of polyunsaturated fatty acids that are present in cell membranes of the brain react as substrates for lipid peroxidation [72]. Moreover, there are rather low levels of glutathione (GSH) in this biological structure, which acts as an endogenous antioxidant in the elimination of ROS. Additionally, some redox-active metals, i.e., iron and copper, profusely subsist in the brain and participate in the catalyzation of ROS formation [52,73]. Furthermore, the central nervous system (CNS) is peculiarly sensitive to oxidative stress, due to the terminal-differentiation characteristics of neurons and weakly antioxidative systems [35]. Consequently, the brain needs an efficient antioxidant system to neutralize the impact of ROS and antiapoptotic mechanisms to support neuronal integrity [61,73]. 

Importantly, it was suggested that various neurons have diver levels of sensitivity to OS, and the amygdala, hippocampus, and cerebellar granule cells have been found to be the most susceptible to this factor, and they are considered to be the first that undergo functional decline [64]. 

Post-mortem brain tissue studies of PD patients showed that the formation of ROS and impaired mitochondrial function are involved in the apoptotic episode in dopaminergic neurons [61]. High oxidation of DNA and proteins, higher levels of lipid peroxidation, as well as the reduction of glutathione were observed during examinations [67]. Therefore, it would be beneficial to diagnose the PD patients before motor manifestations become apparent and, thus, the biomarkers may be useful in the diagnosis and tracking of the disorder [62]. 

Thus far, urate in serum or plasma was found to be a useful progression biomarker, since lower concentrations were diagnosed in patients with PD and ALS [74]. Increased blood concentrations of 8-OHdG, MDA, nitrite, and ferritin, as well as decreased blood levels of catalase, uric acid, glutathione, and total-cholesterol, have been found in patients with PD when compared to healthy individuals [75]. 

Additionally, the scientists revealed that heme oxygenase-1 (HO-1) might reflect further aspects of PD pathology, including oxidative stress, neuroinflammation, mitochondrial damage, and the dysregulation of iron metabolism [76]. HO-1 is mainly an intracellular protein that is related to heme catabolism under the conditions of OS. Nevertheless, it has been detected in extracellular compartments, including cerebrospinal fluid and plasma, with changing levels reflecting disease states [76].

## 5. Salivary Redox Biomarkers in Patients with Neurodegenerative Diseases 

NDDs are clinically, biochemically, and molecularly heterogeneous diseases. Therefore, there is a lack of laboratory biomarkers that allow for the reliable diagnosis of the disease in their asymptomatic stage. In addition, accurate diagnosis of NDDs can often be made *post mortem* [47,48,49,50]. Therefore, alternative diagnostic methods (especially laboratory biomarkers) are still being sought. Redox parameters are increasingly proposed as diagnostic/prognostic biomarkers for neurodegenerative diseases, given the critical role of oxidative stress in NDDs pathogenesis. Interestingly, increasing data indicate a relationship between cerebral (brain) and central (blood) redox homeostasis [52,53,66,73,77,78].

Among many biological fluids, saliva stands out with its unique advantages. It is a non-invasive, easy to collect, non-infectious, and cheap biofluid that reflects the composition of plasma or CSF. Indeed, many studies have shown that the composition of some substances in saliva correlates with their content in the blood or cerebrospinal fluid (e.g. uric acid, creatinine, urea, and tau protein) [1,24,25,26,28,30]. Among these are also antioxidants and cellular oxidation products (e.g. GSH, AGE, AOPP, or MDA), which indicates the use of salivary redox biomarkers in the diagnosis of NDDs. These compounds can pass into saliva by passive/active diffusion and ultrafiltration and they may be an indicator of central oxidation-reduction balance [1,24,25,26,28,30].

Recent studies indicate the use of salivary redox biomarkers, such as oxidatively modified nucleic acids, lipids, or proteins, as well as antioxidants in non-invasive diagnostics of NDDs (Table 1). 

### 5.1. Protein Oxidation Products 

Proteins are the main target of free radical-induced damage in the cell. ROS and RNS oxidize the polypeptide chain of proteins, as well as amino acid residues. This can lead to the disruption of the polypeptide chain, to the formation of cross-links within the same or several polypeptide chains, and to the appearance of altered amino acid residues [72].

AGE, AOPP, Amadori products, protein carbonyls (PC), as well as total thiols were used to show oxidative damage to proteins in patients with PD and dementia, including individuals with Alzheimer’s disease, vascular dementia, mixed dementia, and mild cognitive impairment [25,26,76,79]. 

The researchers observed a statistically significant increase of AGE and AOPP in stimulated and nonstimulated saliva of dementia patients in comparison to the controls [25,26]. Moreover, the levels of both markers were much higher in severe dementia sufferers than in those with mild to moderate dementia [26]. The same pattern could be observed with Amadori products [26]. Nevertheless, protein carbonyls had a higher level in nonstimulated and stimulated saliva of patients with severe, as well as mild to moderate dementia than in controls, in the Polish population. PC was increased in stimulated saliva of individuals with severe than mild to moderate dementia [26]. In the Canadian study, the researchers have not found statistically significant differences among individuals with Alzheimer’s disease and mild cognitive impairment, and control group [79].

Klimuk et al. examined protein glycoxidation products in patients with severe and mild to moderate dementia. as well as in individuals from the control group [26]. The researchers found out that tryptophan, kynurenine, N-formylkynurenine, and dityrosine fluorescence were significantly higher in the stimulated saliva of both subgroups of dementia patients than in controls as well as in the group of individuals with severe dementia in comparison to those with mild to moderate dementia. Moreover, only tryptophan fluorescence was statistically elevated in unstimulated saliva in both subgroups of individuals with dementia in comparison to the control group, whereas the results were divergent in the case of other parameters.

It is believed that oxidized proteins may show a reduction or complete lack of biological activity and tend to form aggregates. This promotes the accumulation of altered proteins in the cell and, on the basis of positive feedback, intensifies further overproduction of ROS [65,72].

### 5.2. Lipid Peroxidation Products 

Lipid peroxidation markers play a vital role in an assessment of brain damage, because this organ is characterized by high lipid composition as well as high oxygen consumption [52,80]. 

The research performed in dementia patients (Alzheimer’s disease, vascular dementia, and mixed dementias) showed an increased level of 8-isop in both stimulated and unstimulated saliva from the study group in comparison to controls [25].

Pena-Bautista et al. have measured a new set of lipid peroxidation products in the saliva samples from dementia patients (Alzheimer’s dementia, frontotemporal dementia, and vascular dementia) while using UPLC/MS-MS (ultra-performance liquid chromatography-tandem mass spectrometry) [80]. These biomarkers include neuroprostanes, such as F2-IsoPs, 4-NeuroPs, dihomo-IsoFs, F2-dihoo-IsoPs, as well as prostaglandins. Although they were found previously in urine and plasma of dementia patients [81,82], UPLC-MS/MS analysis for saliva showed suitable sensitivity, as well as high precision and accuracy. Thus, neuroprostanes can be evaluated, not only in the blood and urine, but also in saliva. Neuroprostanes are stable products of non-enzymatic cyclooxygenation of polyunsaturated fatty acids. They are considered as non-invasive biomarkers of brain damage.

It is believed that lipid peroxidation products (including 8-isop) can react with DNA and proteins, being responsible for disturbances in gene expression/protein synthesis, as well as interfering with many metabolic processes (e.g., uncoupling of oxidative phosphorylation) [52,80].

### 5.3. Nucleic Acid Oxidation Products 

We have found only one study describing the marker of DNA oxidative damage in the saliva of NDDs sufferers [25]. Interestingly, the authors revealed a significantly higher level of 8-OHdG in the stimulated and unstimulated saliva of study group than in controls. 

DNA oxidative injury is particularly dangerous, because it can cause genetic mutations, damage to mitochondrial DNA (mtDNA), and is also responsible for the death of neurons and glial cells [65,72].

### 5.4. Amyloid β 

Amyloid β highly up-regulates NADPH oxidase (NOX), producing a large amount of superoxide anions and other free radicals. Hence, Aβ is one of the ROS sources in the cell [73]. Amyloid β is produced in detectable amounts in various organs [82,83,84], and the identification of the source is essential in determining the clinical utility, as was emphasized in other papers. This protein is deposited in the brain as well as peripheral regions, such as lingual and lacrimal glands and nasal mucosa [25,85]. 

The accumulation of this neurotoxic protein in the secretory epithelium of salivary glands presumably disturbs the local redox balance and it is accountable for the impairment of the structure as well as for the dysfunction of these organs [25,85]. The researchers revealed an increased level of salivary Aβ_42_ levels in AD patients in comparison to the control groups [83,85]. However, Bermejo-Pareja et al. did not show any statistical differences in the saliva concentration of Aβ_42_ between patients with PD and healthy controls [85]. Moreover, the author found unchanged levels of Aβ_40_ between AD patients and healthy subjects.

### 5.5. Antioxidant Defense 

The antioxidant defense in individuals suffering from NDDs is decreased, as shown in the literature. The activity of catalase, salivary peroxidase, level of total antioxidant capacity, and reduced glutathione were significantly lower in both the stimulated and nonstimulated saliva samples from patients with dementia than in the control groups [25,26]. Moreover, some of the compounds (CAT, GSH, TAS, and Px) were also decreased in stimulated and unstimulated saliva from individuals with severe dementia more than in those with mild to moderate dementia. Nonetheless, the total oxidant status (TOS) and oxidative stress index (OSI) were much higher in the saliva of dementia patients than the control group. This indicates that the antioxidant reserves are depleted, and the redox balance is shifted in favor of the oxidation reactions.

It should be assumed that the impaired antioxidant barrier in NDDs is a direct cause of greater oxidative damage to salivary proteins, lipids, and nucleic acids. Indeed, Klimiuk et al. [26] showed that, in nonstimulated saliva from dementia patients, GSH concentration correlated negatively with N-formylkynurenine fluorescence. They also demonstrated a negative correlation between the GSH and AGE of dementia patients. Glutathione is considered to be the most important of the brain antioxidants [65,73] and, therefore, disturbances in its metabolism may result in increased oxidation of cellular biomolecules.

### 5.6. HO-1

HO-1 inhibits ROS production/induction of apoptosis by removing heme from the cell [76].

In individuals with Parkinson’s disease, a significant elevation of salivary HO-1 concentrations was noted in comparison to the healthy controls [76]. This indicates the body’s adaptive response to increased ROS production in patients with PD. ELISA and western blotting both confirmed the usefulness of HO-1 determination in saliva. Interestingly, no changes in HO-1 protein concentration were found, depending on age, gender, L-dopa equivalence, and other comorbidities. However, further research is needed to demonstrate whether the salivary HO-1 level depends on genetic risks for the disease.

## 6. Differences and Similarities in Levels of Biomarkers in Saliva and Blood

Saliva might be used for examination of the levels of different substances and biomarkers since it is easily accessible, and its collection is considered as a non-invasive technique. In comparison to taking other biological fluid samples, as a potential indicator of disease neuropathology, the obtaining of saliva samples can be done with minimal stress for the patient. Therefore, research is being carried out to evaluate whether the levels of biomarkers in saliva may reflect their concentrations in other body fluids.

Choromanska et al. suggested that variations in salivary redox homeostasis are independent of systemic changes (erythrocytes/plasma) in moderate dementia. There was no correlation between salivary redox biomarkers and their content in the blood. The authors revealed that in both saliva/plasma of dementia individuals, only total oxidant status (TOS) was significantly higher in comparison to that of the healthy controls [25]. Moreover, the results showed that the mean concentrations of AOPP, 8-OHdG, and 8-isop and fluorescence of AGE in stimulated and non-stimulated saliva from dementia patients were significantly higher than those from the control group, whereas, in the plasma of dementia patients, only the mean value of AGE was considerably higher than that in healthy individuals. The decreased antioxidant properties of saliva might indicate a higher susceptibility of the salivary glands to oxidative damage, and the oral cavity is much more prone to diseases, while, the increase in SOD and GPx activity in erythrocytes shows the central adaptive response of the body to excessive production of ROS, as it was explained. Therefore, the changes in redox balance within salivary glands are different from systemic ones in people with dementia. However, they showed that the assessment of salivary AGE could be one of the non-invasive biomarkers in diagnosing dementia. Indeed, AGE content in saliva correlated with its plasma level.

However, Klimiuk et al. indicated that both blood and salivary oxidative stress increased with the severity of the disease, and the content of most biomarkers in saliva reflects their blood levels. [26]. The authors showed the very high diagnostic usefulness of salivary and plasma SP/GPx, Amadori products, and GSH in differentiating patients suffering from severe dementia from those with a mild and moderate stage of the disease [26]. The authors found severe oxidative damage to proteins (↑Amadori products, ↑AOPP, ↑PC, ↑AGE, ↑protein glycoxidative modifications, and ↓total thiols) in stimulated and unstimulated saliva, as well as plasma of dementia patients in comparison to the control group. It was emphasized that the degree of protein damage increased along with cognitive dysfunction in individuals with various types of dementia. Interestingly, the concentration of Amadori products in unstimulated saliva was correlated with their level in blood plasma and cognitive impairment in the MMSE (Mini Mental State Examination) scale. Therefore, this biomarker might be used in psychiatric laboratory medicine. However, the levels of GSH in unstimulated and stimulated saliva, as well as plasma, were significantly lower in individuals with severe dementia when compared to those with the moderate stage of the disease and healthy ones. Moreover, the concentration of reduced glutathione (GSH) in unstimulated saliva correlated with the concentration of this marker in blood plasma, which also indicates the potential use of saliva as an alternative diagnostic material to blood [26]. 

The use of Aβ_42_ from saliva as a biomarker for AD is currently innovative in comparison to more traditional detection methods, such as blood studies, cerebrospinal fluid, or imaging [80]. Interestingly, the significance of Aβ levels in saliva in relation to the accumulation of this biomarker in the brain is still unknown, but their concentrations are approximate with those that were obtained from tissues other than the brain, e.g., lens [85,86].

At present, pTau and Aβ_42_ seem to be the best-validated CSF biomarkers, since their sensitivity and specificity are reported at approximately 90–95% for the diagnosis of AD [84]. Additionally, several substances have been examined as possible biomarkers in plasma. However, none of these biomarkers had enough specificity or sensitivity to diagnose AD.

Bermejo-Pareja et al. found that the plasma levels of Aβ_40_ and Aβ_42_ did not differ significantly between patients with AD and control groups, and Spearman rank analysis of plasma and saliva levels was not statistically significant for both marker levels [85].

## 7. Biomarkers and Stage of the Disease

The molecular biomarkers for identifying, as well as classifying, neurodegenerative diseases would be useful because they could aid in performing epidemiological screening, confirming the diagnosis, predicting the outcome of the disease, and identifying distinct groups of sufferers [85]. Additionally, such substances should be advantageous in the monitoring of the disorder’s progression and its sensitivity to treatment [85].

Klimiuk et al. revealed that the levels of CAT, GSH, TAS, and Px were decreased, whereas AOPP, Amadori products, AGE, N-formylkynurenine, as well as tryptophan and kynurenine fluorescence were increased in the stimulated and nonstimulated saliva of patients with severe dementia in comparison to those with mild to moderate stage of the disorder [26]. On the other hand, the PC level was higher in the unstimulated saliva from individuals with severe dementia than in mild to moderate dementia. Additionally, the authors found that dityrosine fluorescence was increased in the stimulated saliva of individuals with severe dementia (0–10 MMSE) in comparison to milder stages (11–23 MMSE) of the disease. In receiver operating characteristic (ROC) analysis, they showed that salivary GSH clearly distinguishes patients with severe dementia from those suffering from mild or moderate dementia (area under the curve (AUC) = 1; sensitivity = 100%; specificity = 100%) [26]. Additionally, salivary GSH positively correlated with GSH concentration in plasma and, therefore, it can be used in non-invasive diagnostics of cognitive impairment. Additionally, Choromanska et al. [25] showed a very high diagnostic value of AGE determination in nonstimulated saliva from patients with moderate dementia compared to the control group (AUC = 0.85, *p* < 0.0001). Interestingly, the decrease in cognitive function in the MMSE scale was associated with an increase in the AGE content in dementia patients.

The research concerning PD sufferers also showed differences in the level of HO-1 in various stages of the disorder [76]. An increase in salivary logHO-1 concentrations was observed in stage 3 in comparison to stage 1 of the disease. Interestingly, ROC analysis also confirmed the diagnostic usefulness of salivary HO-1. It has been shown that salivary HO-1 distinguishes patients with early-stage of PD from the control group (AUC = 76%; sensitivity = 75%; specificity = 70%). Additionally, it should be emphasized that the proposed test is sensitive in the earliest PD stages when a diagnosis of normal/pathological neuromotor aging is the most challenging [76]. 

Moreover, Bermejo-Pareja et al. showed that significant and reproducible levels of salivary Aβ_42_ could be detected in patients with AD. They also demonstrated a specific correlation between salivary Aβ_42_ and the development of AD neuropathology [85]. Interestingly, there were higher levels of salivary Aβ_42_ in mild AD patients in comparison to those with a severe stage of the disorder, who had similar levels of this biomarker to those that were observed in the control group. Moreover, an increase in salivary Aβ_42_ level was noted in older AD patients. 

## 8. Limitations of Salivary Redox Biomarkers

It must be remembered that different factors might affect the quantity as well as the quality of saliva that was collected for examination [87]. In older people, hyposalivation (the reduction of unstimulated salivary flow rate below 0.2 mL/min.) is very often observed [25,26,88]. This might impede saliva collection and limits the use of saliva as diagnostic material. Therefore, in order to eliminate the effect of hyposalivation, parameters evaluated in saliva should be standardized for total protein content or salivary flow rate. However, in elderly people, decreased salivation mainly concerns the submandibular salivary glands. Therefore, this indicates the potential use of stimulated saliva in non-invasive laboratory diagnostics. Indeed, it is well known that stimulated saliva is mainly the secretion of the parotid glands. In the analyzed articles, only in the study of Choromańska et al. [25] and Klimiuk et al. [26] salivary redox biomarkers were standardized for the total protein content. In patients with dementia, the authors observed a decrease in total protein concentration and a reduction of saliva secretion as compared to controls [25,26].

Many drugs can also affect the quantitative and qualitative composition of saliva [89]. These include antihypertensives, antihistamines, analgesics, and chemotherapeutics. Patients taking drugs that affect the central nervous system, such as anxiolytics, neuroleptics, hypnotic, and anti-epileptic drugs, as well as, in particular, tricyclic antidepressants, also suffer from hyposalivation [90]. Indeed, some of these drugs may interact with the cholinergic muscarinic receptors of the salivary glands, while others act on electrolyte transporters, which also reduces salivary secretion. Drugs, such as captopril or metformin, may also change the antioxidant properties of saliva. In addition, polypragmasia and polypharmacotherapy significantly enhance disturbances of salivary gland function [89,90].

Periodontal disease and oral mucosa disorders are the main sources of oxidative stress in the oral cavity [28,33,34]. Therefore, the redox biomarkers should not be used in patients with oral inflammation. 

It is well known that oxidative stress is inextricably linked to age. This process is dependent on the efficiency of enzymatic and non-enzymatic antioxidants, as well as on the rate of ROS formation in the biological systems, according to the free radical theory of aging [91]. Indeed, disturbances in the antioxidant barrier, as well as increased levels of oxidative damage products, have been shown in different tissues of elderly people, such as blood, brain, liver, as well as saliva [88]. Although age can affect the salivary redox biomarkers, it is believed that oxidative stress is enhanced under the influence of NDDs [25,26]. However, studies on a human model with age and gender-matched control group may confirm the usefulness of salivary redox indicators in the diagnosis of NDDs [25,26]. 

Various systemic diseases may also affect the central/salivary redox homeostasis [88]. Indeed, as was shown in recent studies, it is a particularly common problem in the elderly population. Disturbances in the salivary antioxidant barrier that are caused by dysfunction of the salivary glands were observed in patients with diabetes, Sjogren’s syndrome, psoriasis, as well as rheumatoid arthritis [22,28,87,92].

It has been demonstrated that physical exercise, different xenobiotics (tobacco smoke, ethanol, drugs), dental treatment, dental materials, as well as food (chronic high-fat/high-protein diet) that might induce oxidative stress [21,28,30,33,93,94]. This fact is not surprising because the oral cavity is the only place in the body that is exposed to so many environmental factors [33]. 

Needless to say, that collection time of saliva is significant since diurnal variations of specific components were observed, such as, e.g., protein carbonyls or cortisol. Additionally, the way of saliva sampling, its storage, handling, and processing, as well as analysis techniques, are of great importance [87]. Therefore, there is a need to standardize existing saliva collection protocols and develop reference values for salivary redox biomarkers.

On the other hand, original papers concerning salivary biomarkers in selected neurodegenerative diseases showed that the results of researches that were carried out on a limited number of patients, as presented in this review. It would be beneficial to perform further replication of the presented analysis with the use of a larger sample size.

## 9. Other Salivary Biomarkers NDDs

The use of saliva in the diagnosis of NDDs does not only include biomarkers of oxidative stress. Other salivary biomarkers for diagnosis of neurodegenerative diseases involve tau protein, acetylcholinesterase (AChE), and lactoferrin (Table 2).

An aggregated and phosphorylated isoform of tau is one of the components of neurofibrillary tangles in AD. This protein is rapidly degraded in the blood and cerebrospinal fluid, which is why interest in salivary tau protein is increasing. The salivary tau protein can come from several sources. Similarly to Aβ, it can be a filtrate from the blood or be released from the cells of the mucosa lining of the oral cavity [95]. The expression of tau mRNA has also been demonstrated in salivary glandular cells [96] and nerves innervating the salivary glands [97]. The concentration of salivary phosphorylated tau (p-tau) and total tau protein (t-tau) were examined in four studies, in a total of 181 patients with AD. Shi et al. [97], while using the ELISA tests, showed an elevated (*p* < 0.05) p-tau/t-tau ratio in AD patients. Pekels et al. [98] quantified the p-tau/t-tau ratio at various phosphorylation sites while using Western-blot. This study demonstrated upregulated (*p* < 0.05) phosphorylation sites of S396, S404, T404, and the combination of S400 and T403. However, these authors did not prove the existence of a relationship between salivary p-tau/t-tau ratio and brain atrophy or CSF p-tau/t-tau ratio. What is more, this study demonstrated no changes in P-tau181 in the saliva of AD patients, and the wide range of obtained results suggests that this method cannot be useful as a diagnostic method. The other two studies using the ELISA [99] and single molecule array (SIMOA) [100] showed no significant differences in salivary p-tau and t-tau between AD patients and the control group.

Acetylcholinesterase (AChE) inhibitors (AChE-I) are the first-line drugs that are prescribed for symptom management in AD patients. Their use results in the release of acetylcholine (ACh) into the synapse cleft. Salivary acetylcholine activity was researched in three studies, in a total of 66 patients. All three studies used Ellman’s colorimetric method. Sayer et al. [101] demonstrated decreased activity of AChE in the saliva of AD patients vs control (*p* < 0.05), as well as correlation with age in healthy controls (*p* < 0.001). What is more, these authors showed that, within AD patients, AChE activity is reduced in AChE-I non-responders vs AChE-I responders. Bakhtiari et al. [102] and Boston et al. [103] demonstrated the lack of significant changes in AChE salivary activity between AD patients and the control group.

It was demonstrated that AD could be initiated by bacterial or viruses infection of the brain [104]. Lactoferrin (LF), an Aβ binding protein, is one of the main antimicrobial peptides present in the saliva. It is a Fe^3+^ iron-binding glycoprotein with molecular weight, which is associated with its bacteriostatic effect [105]. The bactericidal effect of LF is associated with the N-terminal region that is responsible for destroying the outer cell membrane of Gram-negative bacteria [106]. Evidence suggests that lactoferrin is up regulated in AD brain and, what is more, it might be responsible for the deposition of Aβ [107]. Contrary to expectations, Carro et al. [108], using an initial MS discovery and validation by ELISA, showed a decrease in lactoferrin concentration in the unstimulated saliva from AD patients vs control (*p* < 0.001). Moreover, these studies showed a positive correlation with CSF Aβ42 and t-tau (*p* < 0.001) as well as a positive correlation with minimal state examination in AD patients vs amnestic mild cognitive impairment individuals (aMCI) (p <0.001). This study proved that apparently healthy participants in the control group with low levels of salivary lactoferrin were at a high risk of a MCI development, and even AD in the future.

Another group of researchers, while using proton NMR spectroscopy, found an increased level of propionate in the saliva of AD patients vs control [109]. Liang et al. [110] using UPLC-MS, identified higher concentration of spinganine-1-phosphate, ornithine and phenyllactic acid (*p* < 0.01), as well as lower concentration of inosine, 3-dehydrocarnithine, and hypoxantine (*p* < 0.01) in the saliva of AD patients vs control.

Finally, Lau et al. [99] found no changes in the concentration of trehalose in the saliva of AD patients as compared to the control. The study was conducted on a group of 20 patients with AD while using an extended gate ion-sensitive field-effect transistor biosensor (EG-IDFET).

## 10. Summary and Perspective 

Several studies have showed the usefulness of oxidative stress biomarkers that were measured in the blood, plasma, serum, urine, or CSF in the diagnosis of NDDs [77,78,111,112,113,114,115]. However, so far, scarce literature data describe their diagnostic value in saliva. 

At present, new analytical methods are being developed for the diagnosis of biomarkers in salivary samples (e.g. UPLC/MS-MS) [80]. Additionally, researchers are working on the differentiation of various stages or severity of disorders [26]. Indeed, new biomarkers are still being sought that, when collected in a non-invasive manner, could indicate disease, even before its first symptoms appeared. An ideal laboratory biomarker should be reproducibly measured with standardized and widely available methods, also should be easy to interpret and have appropriate sensitivity and specificity. Klimiuk et al. revealed, for the first time, that selected redox biomarkers might be helpful in the differential diagnosis of dementia [26]. The authors observed that salivary and central oxidative stress both increase with the severity of the disease and correlates with a decrease in cognitive functions. The high diagnostic value of salivary antioxidants and oxidation products was also confirmed by ROC analysis [25,26]. Salivary GSH and AGE are particularly noteworthy, presenting high sensitivity and specificity differentiate patients with dementia from healthy control as well as people with mild/moderate dementia from severe dementia. In addition, the GSH and AGE levels in saliva correlate with their plasma content.

Interestingly, salivary HO-1 can be used in noninvasive diagnostics of the early stages of PD [76]. The concentration of HO-1 in nonstimulated saliva was significantly higher in patients with idiopathic PD as compared to non-neurological controls matched for sex. What is essential, this parameter does not depend on age, various comorbidities, as well as medication. ROC analysis has also confirmed its diagnostic usefulness [76].

Early diagnosis of NDDs is vital for establishing the proper treatment of the disorder in sufferers [116]. Therefore, further researches are necessary for this area, since the salivary biomarkers seem to be a promising diagnostic material, because it is easily accessible and non-expensive. Furthermore, salivary redox biomarkers are used in the diagnosis of metabolic diseases [19,20,21,22,23,24,117,118,119,120,121] or cancer [122]. 

**Figure 1:**
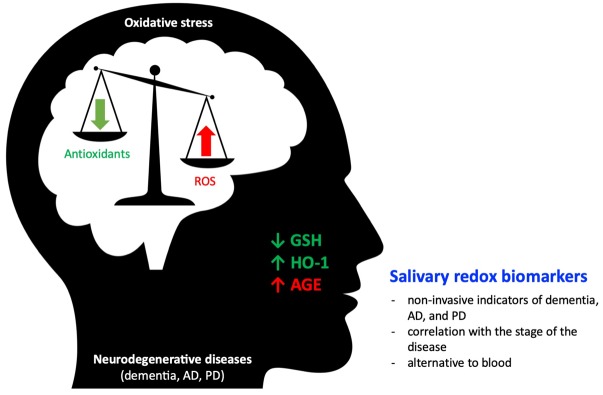
Salivary redox biomarkers in selected neurodegenerative diseases.

Summarizing, the reviewed papers concerning salivary redox biomarkers in NDDs revealed their prospective usefulness in clinical practice (Figure 1). They might be utilized in diagnostics of the disorders as well as indicators of disease progression [26,76]. It was emphasized that saliva might be a promising, easily accessible, and non-expensive, diagnostic tool for oxidative stress biomarkers in patients with neurodegenerative disease. However, further studies are required in larger cohorts. Additionally, there is a need to standardize saliva collection protocols.

## 11. Conclusions

1. Salivary redox biomarkers can be non-invasive indicators of NDDs. The level of many biomarkers in saliva correlates with their plasma content and the severity of NDDs.
2. The protein oxidation products, such as AGE, as well as antioxidant molecules, such as GSH and HO-1, appear to be particularly interesting in NDDs diagnostics.
3. The clinical usefulness of salivary redox biomarkers of NDDs requires further verification in clinical trials on a large population of patients. Additionally, there is a need to standardize saliva collection protocols and develop reference values for salivary redox biomarkers.

## Figures and Tables

**Table 1:** Selected studies included in the review.

Reference	Study Population	Smokers/Periodontal Disease Included	Saliva Collection				Salivary Markers (Analytical Method)	Endpoints
			Type	Time	Centr.	Storage		
Choromanska et al. 2017	study group: 80 patients with moderate (MMSE 11–18) AD, VaD, MxD (mean age 80.12); control group: 80 heathy subjects (mean age 80.12, MMSE >23) age- and sex-matched to the study group	No/No	NWS, SWS	8 AM – 10 AM	3000 x g, 20 min, 4 ^o^C	−80 ^o^C	total protein (colorimetry)	↑ in the study group in both NWS and SWS
							uric acid (UA) (colorimetry)	↓ in NWS of study group (*p* < 0.05)
							CAT (colorimetry)	↓ in NWS (*p* ˂ 0.05) and in SWS (*p* ˂ 0.008) of study group
							Px (colorimetry)	↓ in NWS (*p* ˂ 0.002) and in SWS (*p* ˂ 0.002) of study group
							TOS (colorimetry)	↑ in NWS (*p* < 0.006) and in SWS (*p* < 0.009) of study group
							OSI	↑ in NWS (*p* < 0.01) and in SWS (*p* < 0.02) of study group
							TAC (colorimetry)	↓ in NWS (p<0.02) and SWS (*p* < 0.001) of study group
							AGE (fluorimetry)	↑ in NWS (*p* < 0.03) and in SWS (*p* < 0.02) of study group negative correlation between AGE NWS and cognitive function in MMSE scale (*r* = −0.45, *p* = 0.04) high diagnostic value of AGE NWS in the differentiation of patients with dementia from healthy control (AUC = 0.85, *p* < 0.0001; sensitivity of 75.68% and specificity of 75.86%)
							AOPP (colorimetry)	↑ in NWS (*p* < 0.007) and in SWS (*p* < 0.02) of study group
							8-isop (ELISA)	↑ in NWS (*p* < 0.04) and in SWS (*p* < 0.001) of study group
							8-OHdG (ELISA)	↑ in NWS (*p* < 0.007) and in SWS (*p* < 0.0004) of study group
Klimiuk et al. 2019	study group: 50 patients with SD (0-10 MMSE) and MMD (11-23 MMSE) AD, VaD, MxD (mean age 80.24); control group: 50 healthy subjects 50 (mean age 80.82) age- and sex-matched to the study group	No/No	NWS, SWS	8 AM – 10 AM	3000 x g. 20 min, 4 ^o^C	−80 ^o^C	CAT (colorimetry)	↓ in NWS (*p* < 0.001 in both groups) in SD and MMD patients compared to controls ↓ in NWS of SD compared to MMD patients (*p* < 0.001) ↓ in SWS of MMD (*p* < 0.001) and SD patients (*p* < 0.001) compared to controls ↓ in SWS of SD compared to MMD patients (*p* < 0.001)
							GSH (colorimetry)	↓ in NWS (*p* < 0.001 in both groups) in SD and MMD patients compared to controls ↓ in NWS of SD compared to MMD patients (*p* < 0.001) ↓ in SWS of MMD (*p* < 0.001) and SD patients (*p* < 0.001) compared to controls ↓ in SWS of SD compared to MMD patients (*p* < 0.001) positive correlation between salivary and plasma levels (SD: *r* = 0.45, *p* = 0.002; MD: *r* = 0.51, *p* = 0.01)
							TAS (colorimetry)	↓ in NWS (*p* < 0.001 in both groups) in SD and MMD patients compared to controls ↓ in NWS of SD compared to MMD patients (*p* < 0.001) ↓ in SWS of MMD (*p* < 0.001) and SD patients (*p* < 0.001) compared to controls ↓ in SWS of SD compared to MMD patients (*p* < 0.001)
							Px (colorimetry)	↓ only in NWS of SD compared to controls (*p* < 0.001) ↓ in NWS of SD compared to MMD patients (*p* < 0.001) ↓ in SWS of MMD (*p* < 0.01) and SD patients (*p* < 0.001) compared to controls ↓ in SWS of SD compared to MMD patients (*p* < 0.001)
							AOPP (colorimetry)	↑ in NWS of MMD and SD compared to controls (*p* < 0.001 in both groups) ↑ in NWS of SD than in MMD individuals (*p* < 0.001) ↑ in SWS of MMD and SD patients than in controls (p<0.001 in both groups) ↑ in SWS of SD than MMD patients (*p* < 0.001)
							Amadori products (colorimetry)	↑ in NWS of MMD and SD compared to controls (*p* < 0.001 in both groups) ↑ in NWS of SD than in MMD individuals (*p* < 0.001) ↑ in SWS of MMD and SD patients than in controls (*p* < 0.001 in both groups) ↑ in SWS of SD than MMD patients (*p* < 0.001) high correlation between salivary and plasma levels (SD: *r* = 0.67, *p* < 0.001; MD: *r* = 0.62, *p* = 0.001)
							PC (colorimetry)	↑ in NWS of MMD and SD compared to controls (*p* < 0.001 in both groups) ↑ in SWS of MMD and SD patients than in controls (*p* < 0.001 in both groups) ↑ in SWS of SD than MMD patients (*p* < 0.001)
							AGE (fluorimetry)	↑ in NWS of MMD and SD compared to controls (*p* < 0.001 in both groups) ↑ in NWS of SD than in MMD individuals (*p* < 0.001) ↑ in SWS of MMD (*p* < 0.05) and SD (*p* < 0.001) patients than in controls ↑ in SWS of SD than MMD patients (*p* < 0.001) positive correlation between salivary and plasma levels (SD: *r* = 0.62 *p* < 0.001; MD: *r* = 0.69, *p* < 0.001)
							total thiols (colorimetry)	↓ in NWS of MMD and SD compared to controls (*p* < 0.001 in both groups) ↓ in SWS of MMD and SD compared to controls (*p* < 0.001 in both groups)
							tryptophan (fluorimetry)	↑ in NWS of MMD (*p* < 0.01) and SD (*p* < 0.001) patients compared to controls ↑ in NWS of SD than MMD (*p* < 0.001) patients ↑ in SWS of MMD and SD than in controls (*p* < 0.001 in both groups) ↑ in SWS of SD than MMD (*p* < 0.001)
							kynurenine (fluorimetry)	↑ in NWS only of SD (*p* < 0.001) patients compared to controls ↑ in NWS of SD than MMD (*p* < 0.001) patients ↑ in SWS of MMD and SD than in controls (*p* < 0.001 in both groups) ↑ in SWS of SD than MMD (*p* < 0.001)
							N-formylkynurenine (fluorimetry)	↑ in NWS only of SD (*p* < 0.001) patients compared to controls ↑ in NWS of SD than MMD (*p* < 0.001) patients ↑ in SWS of MMD and SD than in controls (*p* < 0.001 in both groups) ↑ in SWS of SD than MMD (*p* < 0.001)
							Dityrosine (fluorimetry)	↑ in NWS of MMD (*p* < 0.001) patients compared to controls ↓ in NWS of SD patients (*p* < 0.001) ↑ in SWS of MMD and SD than in controls (*p* < 0.001 in both groups) ↑ in SWS of SD than MMD (*p* < 0.001)
Pena-Bautista et al. 2019	study group: 30 patients with AD, FTD, VaD (age range 50–75)	Yes-21%; Former smoker (>10 years)- 15%/No	SWS	10 AM – 12 AM	3500 x g, 10 min, 4 ^o^C	-80 ^o^C	IsoPs, IsoFs, NeuroPs, NeuroFs (UPLC-MS/MS)	new set of lipid peroxidation biomarkers (neuroprostanes) was measures for the first time in saliva samples, including F2-IsoPs, 4-NeuroPs, prostaglandins, dihomo-IsoFs, F2-dihoo-IsoPs; UPLC-MS/MS showed suitable sensitivity, as well as high precision and accuracy to be applied to saliva samples from NDDs patients; methodology was validated and showed high-throughput, satisfactory precision [coefficients of variation 2–11% (intra-day) and 5–12% (inter-day)], and high sensitivity (limits of detection 0.02–2 nmol L^−1^); reliability of the presented method was evaluated by analysis of samples of spiked saliva, and the recoveries were 80-120% for most of the analytes
Su et al. 2008	study group: 15 patients with AD (mean age 82.40), 21 patients with MCI (mean age 81.14); control group: 30 healthy subjects (mean age 69.20)	No/No	NWS	8 AM – 10 PM (at 8AM, 10AM, 2PM, 4PM, 10PM)	10,000 x g, 20 min, 4 ^o^C	−80 ^o^C	PC (colorimetry)	no statistically significant differences among the diagnostic groups; diurnal variation in AD and MCI patients as well as controls (peak of salivary PC concentrations at 2PM); repeat multivariate analyses revealed that overall mean protein carbonyl concentrations were not different (*p* = 0.45) between the ApoE ɛ4 noncarriers (2.0 ± 0.20 nmol/mg protein) and carriers (1.71 ± 0.32 nmol/mg protein). While, salivary carbonyl levels varied significantly (*p* < 0.0001) as a function of collection time (peak values at 2 PM)
Song et al. 2018	Study group: 58 patients with early idiopatic PD (mean age 70.83); control group: 59 healthy subjects (mean age 66.74)	No/Yes	NWS	N.A (not available)	10,000 rpm), 20 min., 4 ^o^C	–80 ^o^C	HO-1 (ELISA, western blotting)	densitometric analysis of the salivary HO-1 bands relative to AMY1A (internal control) revealed a significant elevation of HO-1 protein concentrations in PD individuals compared to controls (*p* = 0.0014); ↑ salivary HO-1 levels in PD patients than in controls (*p* = 0.03); ↑ salivary logHO-1 concentrations in PD patients in H & Y stage 1 (early PD) than in controls (stage 0; *p* = 0.0006); difference statistically significant (*p* = 0.004) noted between PD stages 1 and 3 (↑ salivary logHO-1 concentrations in stage 3 than stage 1 of PD patients); salivary HO-1 concentrations distinguish PD subjects with early-stage of the disease (H & Y stage 1) from non-PD controls (H & Y stage 0) with an area under the curve of 76% (95% CI: 63–90); at the arbitrary cut-off of 4.5 ng/mL, sensitivity was 75% (95% CI: 54–96) and specificity amounted to 70% (95% CI: 5881) for H&Y stage 1 versus controls
Sabbagh et al. 2018	study group: 15 patients with AD (mean age 77.8, mean MMSE 19.0); control group: 7 healthy subjects (mean age 60.4, mean MMSE 29.0)	N.A/No	NWS	N.A	N.A	N.A	β amyloid (Aβ_42_) (ELISA)	↑ salivary Aβ_42_ levels in AD patients than in controls (51.7± 1.6 pg/mL for AD patients and 21.1±0.3 pg/mL for controls, *p*<0.05); intra assay coefficient of variation (CV) was 3.10 for AD and 1.34 for controls; more development is required, including multi-laboratory validation, test-retest validity, and identification of confounders of diurnal variations; given the strength of the results from the study of Sabbagh et al., salivary Aβ42 warrants further investigation as a potential biomarker for mild to moderate AD
Bermejo-Pareja et al. 2010	study group: 70 patients with AD (mean age 77.20, mean MMSE 17); study group: 51 patients with PD (mean age 72.96, mean MMSE 28); control group: 56 elderly nondemented controls without neurological disease or cognitive impairment (mean age 74.35)	N.A./Yes	NWS	1 PM	1500 rpm,5 min.	−80 ^o^C	β amyloid (Aβ_40_, Aβ_42_) (highly sensitive ELISA)	↑ salivary Aβ_42_ in AD patients compared with PD and control groups (not statistically significant); ↑ salivary Aβ_42_ levels in mild AD patients; whereas the severe AD stage, had similar level than those observed in control group; no differences in saliva concentration of Aβ_42_ between patients with PD and healthy controls; unchanged Aβ_40_ between AD patients and healthy subjects; unchanged saliva Aβ_40_ expression within all the studied samples; ↑ ratio between saliva Aβ_42_ and Aβ_40_ (but not statistically significant) in mild and moderate AD patients in comparison to control subjects, whereas it was unchanged in severe AD patients; ↑ salivary Aβ_42_ in older AD patients; association between saliva Aβ_42_ levels and AD was independent of established risk factors, including age or Apo E, but was dependent on sex and functional capacity; ↑ levels of Aβ_42_ (not statistically significant) in patients with AD and without the Apo E ε4 allele in comparison to those with the allele; levels of Aβ_42_ were similar in controls with and without Apo E ε4 allele; levels of Aβ_40_ and Aβ_42_ in plasma did not differ significantly between AD patients and controls (259 ± 91.9 pg/mL vs. 225.1 ± 77.3 pg/mL, and 42.4 ± 92.7 pg/mL vs. 52.4 ± 68.9 pg/mL, respectively); Spearman rank analysis of saliva and plasma levels was not significant for Aβ_40_ as well as Aβ_42_ levels; authors showed the remarkable reproducibility of the saliva Aβ in different series of repetitive measurements

**Table 2:** Other salivary biomarkers used in the neurodegenerative diseases (NDDs) diagnostics.

Reference	Study Population	Salivary Biomarker	Analytical Method	Endpoints
Lau et al. 2015	20 patients with AD, 20 with PD, and 20 healthy controls	A*β*42 t-tau p-tau trehalose	ELISA, EG-IDFET biosensor	↑ trehalose and ↑ p-tau *vs* controls A*β*42 not detected t-tau unchanged
Shi et al. 2011	21 patients with AD and 38 healthy controls	A*β*42 t-tau p-tau	ELISA (Luminex assay)	A*β*42 not detected ↑t-tau and ↑p-tau *vs* controls p < 0.05
Ashton et al. 2018	53 patients with AD and 160 healthy controls	t-tau	Single molecule array (SIMOA)	t-tau unchanged
Pekels et al. 2019	46 patients with AD and 47 healthy controls	t-tau p-tau	Western Blot	↑ p-tau, ↑t-tau at phosphorylation site S396, S404, S400, T403, T404 *vs* controls
Bakhtiari et al. 2017	15 patients with AD and 15 healthy controls	AChE activity	Ellman’s colorimetric method	AChE activity unchanged
Boston et al. 2008	15 patients with AD and 13 healthy controls	AChE activity	Ellman’s colorimetric method	AChE activity unchanged
Sayer et al. 2004	22 AD responders to AChE inhibitor, 14 AD no responders to AChE inhibitor, 11 healthy controls	AChE activity	Ellman’s colorimetric method	↓ AChE activity in the saliva of AD no responders *vs* controls (*p* < 0.005) AChE activity in the saliva of AD responders and AD no responders did not statistically different
Carro et al. 2017	80 patients with AD and 91 healthy controls	lactoferrin	ELISA	↓ lactoferrin in the saliva of AD patients *vs* healthy controls (*p* < 0.001)
Liang et al. 2015	256 patients with AD and 218 healthy controls	sphinganine-1-phosphate ornithine phenyllactic acid inosine 3-dehydrocarnitine hypoxantine	UPLC-MS	↑ sphinganine-1-phosphate,↑ ornithine, ↑ phenyllactic acid ↓ inosine, ↓3-dehydrocarnitine ↓ hypoxantine in the saliva of AD *vs* controls (*p* < 0.01)

## Abbreviations

↑	higher
↓	lower
•OH	hydroxyl radical
3-NT	3-nitrotyrosine
4-HNE	4-hydroxy-2-nonenal
8-isop	8-isoprostanes
8-OHdG	8-hydroxy-2’- deoxyguanosine
8-OHG	8-hydroxyguanosine
AD	Alzheimer’s disease (dementia)
AGE	advanced glycation end products; AOPP – advanced oxidation protein products
ALS	amyotrophic lateral sclerosis
Aβ	amyloid-β
CAT	catalase
CG	control group
CNS	central nervous system
DLB	dementia with Lewy bodies
DNA	deoxyribonucleic acid
ER	endoplasmic reticulum
EWSR1	Ewing sarcoma RNA-binding protein 1
FTD	frontotemporal dementia;
FUS	fused-in sarcoma
GPx	glutathione peroxidase
GSH	glutathione
GSH	reduced glutathione
H_2_O_2_	hydrogen peroxide
HBV	hepatitis B virus
HCV	hepatitis C virus
HD	Huntington’s disease
HIV	human immunodeficiency virus
HO-1	Heme oxygenase-1
hs-CRP	high-sensitivity C-reactive protein
LTP	long-term potentiation
MCI	mild cognitive impairment
MDA	malondialdehyde
MMD	mild to moderate dementia (MMSE 11-23);
MMSE	Mini Mental State Examination
MxD	mixed dementias
NDDs	neurodegenerative diseases
NFTs	neurofibrillary tangles
NO	nitric oxide
Nox	nicotinamide adenine dinucleotide phosphate NADPH) oxidase
NWS	non-stimulated saliva
O_2_•	superoxide anion
ONOO	peroxynitrite
OS	oxidative stress
OSI	oxidative stress index
PC	protein carbonyls
PD	Parkinson’s disease
PrP	prion protein
pTau	tau protein
PUFA	polyunsaturated fatty acid
Px/SP	salivary peroxidase
RNA	ribonucleic acid
RNS	reactive nitrogen species
ROS	reactive oxygen species
SD	severe dementia (MMSE 0-10)
SG	study group
SOD	superoxide dismutase
SWS	stimulated saliva
TAC	mean total antioxidant capacity
TAF15	TATA-binding protein-associated factor15
TAS	total antioxidant status
TBARS	thiobarbituric acid reactive substances
TDP-43	transactive response (TAR) DNA-binding protein 43
TOS	mean total oxidant status
TR	thioredoxin reductase
UA	uric acid
UPDRS	Unified Parkinson’s Disease Rating Stage
VaD	vascular dementia
WHO	World Health Organization
XO	xanthine oxidase
